# Colonization of the cervicovaginal space with *Gardnerella vaginalis* leads to local inflammation and cervical remodeling in pregnant mice

**DOI:** 10.1371/journal.pone.0191524

**Published:** 2018-01-18

**Authors:** Luz-Jeannette Sierra, Amy G. Brown, Guillermo O. Barilá, Lauren Anton, Carrie E. Barnum, Snehal S. Shetye, Louis J. Soslowsky, Michal A. Elovitz

**Affiliations:** 1 Maternal Child Health Research Center, Perelman School of Medicine, University of Pennsylvania, Philadelphia, Pennsylvania, United States of America; 2 McKay Orthopedic Research Laboratory, University of Pennsylvania, Philadelphia, Pennsylvania, United States of America; Fred Hutchinson Cancer Research Center, UNITED STATES

## Abstract

The role of the cervicovaginal (CV) microbiome in regulating cervical function during pregnancy is poorly understood. *Gardnerella vaginalis* (*G*. *vaginalis*) is the most common bacteria associated with the diagnosis of bacterial vaginosis (BV). While BV has been associated with preterm birth (PTB), clinical trials targeting BV do not decrease PTB rates. It remains unknown if *G*. *vaginalis* is capable of triggering molecular, biomechanical and cellular events that could lead to PTB. The objective of this study was to determine if cervicovaginal colonization with *G*. *vaginalis*, in pregnant mice, induced cervical remodeling and modified cervical function. CD-1 timed-pregnant mice received a 5X10^8^ CFU/mL intravaginal inoculation of *G*. *vaginalis* or control on embryonic day 12 (E12) and E13. On E15, the mice were sacrificed and cervicovaginal fluid (CVF), amniotic fluid (AF), cervix, uterus, placentas and fetal membranes (FM) were collected. Genomic DNA was isolated from the CVF, placenta, uterus and FM and QPCR was performed to confirm colonization. IL-6 was measured in the CVF and AF and soluble e-cadherin (seCAD) was assessed in the CVF by ELISA. RNA was extracted from the cervices to evaluate IL-10, IL-8, IL-1β, TNF-α, Tff-1, SPINK-5, HAS-1 and LOX expression via QPCR. Mucicarmine and trichrome staining was used to assess cervical mucin and collagen. Biomechanical properties of the cervix were studied using quasi-static tensile load-to-failure biomechanical tests. *G*. *vaginalis* successfully colonized the CV space. This colonization induced immune responses (increased IL-6 levels in CVF and AF, increased mRNA expression of cervical cytokines), altered the epithelial barrier (increased seCAD in the CVF), induced cervical remodeling (increased mucin production, altered collagen) and altered cervical biomechanical properties (a decrease in biomechanical modulus and an increase in maximum strain). The ability of *G*. *vaginalis* to induce these molecular, immune, cellular and biomechanical changes suggests that this bacterium may play a pathogenic role in premature cervical remodeling leading to PTB.

## Introduction

Preterm birth (PTB) is the leading cause of perinatal morbidity and mortality worldwide. In the United States, one in nine babies is born prematurely and over 11 million PTB cases were reported last year [1, 2]. Premature babies have a higher incidence of developing medical complications [3] resulting in a financial cost of over 26 billion dollars a year in the United States alone [1]. Despite ongoing research, there are no effective strategies to predict or prevent the majority of preterm births. Recently, studies focusing on the causes of PTB have shown that women with bacterial vaginosis (BV) are at higher risk for spontaneous PTB (sPTB) [4–6]. BV is the most common genital tract infection affecting women worldwide [7]. This disease is characterized by a polymicrobial imbalance, or dysbiosis, of the natural microflora of the cervicovaginal (CV) space. While studies have shown an association between BV and PTB, [8] clinical trials targeting treatment of BV have failed to show differences in PTB rates [9–11]. The human microbiome project has provided valuable information about the diverse bacteria subspecies that make up a BV-like state [12, 13]. CV microflora mainly composed of *Lactobacilli* subspecies (spp.) is considered to be associated with a healthy CV space [8, 10, 14–24] while the lack of these species is integral to the diagnosis of BV and is considered a marker of an unhealthy CV space. Many BV cases are characterized by a decrease of *Lactobacillus* subspecies (spp.) and an increase in biofilms that may include *Mobilincus* spp., *Mycoplasma hominis*, *Atopobium vaginae*, *Bacterioides* spp. and *Prevotellla* spp. and *Gardnerella vaginalis (G*. *vaginalis)* [25, 26]. The conflicting data regarding BV and sPTB may be due, in part, to the role and/or pathogenicity of the different organisms that may compose the clinical diagnosis of BV [27, 28]. Since *G*. *vaginalis* is predominantly found in most cases of BV [29–31], the ability of this bacterium to induce cellular and molecular changes in the CV space, during pregnancy, is of scientific and clinical interest [27].

Recent studies have shown that disruption of the cervical epithelial barrier appears to be an important primary step critical to the initiation of cervical remodeling [32–34]. Cervical remodeling is a process that starts weeks, if not months, prior to parturition [32, 33, 35]. During this cervical remodeling process, cervical tissue undergoes robust changes at the molecular, histological and biomechanical levels to allow for delivery of a fetus [33, 36]. A clinical study attempting to identify biomarkers for cervical epithelial remodeling found increased levels of soluble epithelial-cadherin (seCAD) within the CV space [37]. seCAD, a soluble byproduct of proteolytic cleavage of e-cadherin (a member of the adherens junction complex), which may act as a physiologically relevant biomarker capable of predicting sPTB.[37]. Additionally, there is enhanced expression of several genes in the mouse cervix near delivery. These genes are responsible for cervical distensibility (Hyaluronan, HA) [38], collagen disorganization (lysyl oxidase or LOX) [39, 40], and initiate changes to the cervical extracellular matrix (trefoil factor I (Tff-1) and a Serine protein inhibitor Kasal 5 (SPINK-5)) [35, 41, 42]. Histology of the mouse cervix has revealed evidence of collagen rearrangement which is consistent with previous studies showing that the stiffness of the cervix decreases as parturition approaches [32]. Concordantly, cervical biomechanical studies in the mouse have provided valuable information to understanding how important structural mechanical properties, such as cervical load and stiffness, change at different embryonic stages [33, 43]. Recently, Barnum *et al* evaluated the intrinsic material properties of the mouse cervix during pregnancy [43]. This study provides evidence that alterations to the biomechanical properties of the cervix are likely part of the process of cervical remodeling.

While some of the processes governing cervical remodeling at term have been revealed, how specific bacteria, associated with BV, modify these processes have not been studied. Therefore, the objective of this study was to determine if specific BV-associated bacteria can be pathogenic during pregnancy and induce premature cervical remodeling. We hypothesize that *G*. *vaginalis* colonization of the CV space alters cervical function, contributes to dysfunction of the cervical epithelial barrier and, consequently, initiates cervical remodeling. We created a humanized pregnant mouse model of *G*. *vaginalis* colonization to determine if *G*. *vaginalis* colonization altered the local immune response, induced cervical remodeling and/or altered cervical biomechanics. Our results provide evidence that *G*. *vaginalis* colonization of the CV space leads to inflammation, cervical remodeling and altered cervical biomechanics.

## Materials and methods

### Animals

CD-1 timed-pregnant mice were purchased from Charles River Laboratories (Wilmington, MA). We considered E0 as mating day and E1 was determined based on presence of copulatory plug. Animals were shipped on day 10 after mating, and housed individually in our facilities. These animals were acclimated for 4 days before performing experiments. All the experiments were performed in accordance with the National Institutes of Health Guidelines on Laboratory Animals and with approval from the University of Pennsylvania’s Institutional Animal Care and Use Committee (IACUC #:805513).

### Bacterial cultivation

*Gardnerella vaginalis* was purchased from the ATCC depository (ATCC# 14019) and grown anaerobically at 37°C with 5% CO_2_ in Tryptic Soy Broth (TSB) (Becton, Dickinson and Company, Sparks MD, USA) or Tryptic Soy Agar (TSA) (Becton, Dickinson and Company, Sparks MD, USA) supplemented with 5% horse serum (Gibco, Thermo Fisher Scientific). Efficient bacteria growth was measured and quantified by colony forming unit (CFU) assays. Bacteria were centrifuged twice to remove the growth media and the final pellet was resuspended in sterile filtered sugar water (10% fructose, 10% maltose, 10% glucose in sterile H_2_O (Sigma-Aldrich, Saint Louis MO, USA)) for use in animal experiments. This sugar water was used as the control in our animal trials.

### Cervicovaginal colonization with *G*. *vaginalis*

We created a pregnant mouse model of *G*. *vaginalis* colonization as follows. CD-1 embryonic day 12 (E12) timed-pregnant mice were anaesthetized with isoflurane and five cervicovaginal lavages were performed with 100 μL of sterile PBS prior to control treatment or bacterial inoculation. Bacterial doses were determined using published data in a non-pregnant mouse model [44], and then recapitulating similar *G*. *vaginalis* loads in pregnant mice. The animals then received an intravaginal inoculation of *G*. *vaginalis* by inserting a sterile pipette tip and injecting 50 μL of 5X10^8^ CFU/ml or sugar water. The inoculations were performed on E12 and repeated on E13 for both the *G*. *vaginalis* and the control group. This time point was chosen to mimic a change in the cervicovaginal microflora early in pregnancy. Immediately post-inoculation, each animal was positioned in dorsal decubitus under isoflurane anesthesia for 3 minutes and 100% pure petroleum jelly (Vaseline, Unilever USA) was added with a sterile swab to ensure the inoculum would remain within the cervicovaginal space. Animals were observed for 48 hours and specimens were collected on E15.

Using this protocol, we performed five separate trials. The first three trials were performed to 1) determine the preterm birth rate and 2) to collect fluids/tissues for assessment of inflammation, cervical remodeling and bacterial colonization. A fourth trial was performed to assess the effects of higher CFU dose, where we increase the bacteria load to 5X10^10^ CFU/mL. A final trial was performed to collect whole cervices for biomechanical testing (N = 12 animals in each experimental group) and cervical histology (N = 4 animals in each experimental group). In the first trial, on E15, a subset of the *G*. *vaginalis* (N = 12) and control (N = 8) inoculated animals were sacrificed to collect tissues for downstream assays (described below). The remaining animals (*G*. *vaginalis*, N = 4 and control, N = 4) were monitored for preterm birth and allowed to deliver to record pup weight and size. Preterm birth was defined as delivery prior to E18. Around 24 hours post-delivery, we counted and weighed individual pups in each litter. For the remaining trials, dams were sacrificed on E15 and the following specimens were collected from both *G*. *vaginalis* (N = 10 per trial) and control (N = 8–12 per trial) groups: cervicovaginal fluid (CVF), amniotic fluid (AF), cervix, lower uterus, placentas and fetal membranes. To assess for active colonization of *G*. *vaginalis*, on E15, immediately following CVF collection (N = 12), 50 μL of CVF was spread on Tryptic Soy Agar supplemented with 5% rabbit blood and incubated for 48 hours under the conditions mentioned above.

### Tissue and specimen collection

From the first three animal trials, on E15 CVF, AF, cervix, lower uterus, placenta and fetal membranes were collected. CVF was collected by gently rinsing the cervicovaginal space (pipetting in and out seven times) with 100 μL of sterile PBS. The washes were pooled together into one sterile tube for each dam. AF was collected by aspirating the fluid out of the fetal sacs with a 19 gauge needle. AF from all pups per dam were collected and pooled into one tube and spun at 1,500 rpm for 5 minutes at 4°C. The AF supernatants were stored at -80°C until needed. The cervix was dissected away from the vagina and the lower uterus and was collected after removing the bladder, adipose tissue and rectum. A total of four placentas and their respective fetal membranes were collected from the four fetuses closest to the cervix. The cervices, lower uterus, placentas and fetal membranes were collected and flash frozen in liquid nitrogen and stored at -80°C until needed for downstream assays.

From the fourth animal trial, cervices were collected and placed in 10 mL of 4% formalin, stored for 48–72 hours and used for tissue histology and staining as described below. For biomechanical testing, a second cohort of mice from the same animal trial were sacrificed and stored at -20°C and cervical tissues were harvested at the time of testing as described below.

### Genomic DNA isolation and QPCR

Genomic DNA (gDNA) was isolated and purified from the CVF with the ZR fecal MiniPrep DNA extraction kit (Zymo Research, Irvine, CA, USA). To purify gDNA from placenta, uterus and fetal membranes we used the DNeasy Blood and Tissue mini column DNA extraction kit (Qiagen, Germantown, MD, USA) following the manufacture’s protocol. To quantify the amount of *G*. *vaginalis* gDNA, we used a 16S specific probe to this bacterium (Applied Biosystems, Foster City, CA, USA). gDNA from the CVF, fetal membranes, uterus and placenta was quantified by QPCR to determine tissue specific colonization. A standard curve was created from serially diluted gDNA from *G*. *vaginalis* to quantify the amplification. This standard curve was used for relative quantification of *G*. *vaginalis* abundance using the 7900HT Real-Time PCR System (Applied Biosystems). The results were analyzed using the RQ manager software v2.4 (Applied Biosystems).

### ELISA assays

Amniotic fluid was used for measurement of IL-6. Cervicovaginal fluid was used for measurement of IL-6 and soluble E-cadherin (seCAD) using commercially available ELISA assay kits following the manufacturer’s protocol (R&D Systems, Minneapolis, MN, USA).

### RNA isolation from cervix

To isolate RNA from the previously collected cervices, we placed the cervix in a round bottom 2.0 mL Eppendorf tube with TRIzol (Invitrogen, Thermo-Fisher Scientific). The cervices were then mechanically homogenized with stainless steel beads (5mm, Qiagen) at a frequency of 30 Hz/sec for 10 minutes in a TissueLyser II (Qiagen) and underwent phenol-chloroform extraction. RNA concentration was determined via a NanoDrop 2000 Spectrophotometer (Nanodrop^™^ Rockland, DE) prior to the generation of cDNA.

### cDNA generation and QPCR

cDNA was generated from 1 μg of isolated RNA from cervical tissue using the high capacity cDNA reverse transcription kit (Applied Biosystems, Thermo-Fisher Scientific). QPCR was performed on the 7900HT Real-Time PCR System (Applied Biosystems) using the TaqMan Universal PCR Master Mix (Applied Biosystems) according to the manufacturers’ protocols. The standard curve method was used for relative expression quantification using the RQ manager software v2.4 (Applied Biosystems). In TaqMan QPCR assays, the relative abundance of the target of interest was divided by the relative abundance of 18S in each sample to generate a standardized abundance for the target transcript of interest. All mRNA primers were purchased from Applied Biosystems: IL-10, IL-8, IL-1β, TNF-α, Tff-1, SPINK-5, HAS-1, LOX and 18S (TaqMan gene expression assays, Applied Biosystems, Thermo-Fisher).

### Trichrome and mucicarmine assay

At E15, post *G*. *vaginalis* inoculation, the cervices were harvested as noted above, and placed in formalin for 48–72 hours and paraffin embedded. The cervices were sectioned (10 μm) and mounted onto glass microscope slides. These sections were stained using hematoxylin and eosin (H&E) (ScyTech, Logan Utah, USA), trichrome and mucicarmine staining kits (Abcam, Cambridge, MA, USA), following the manufacturer’s instructions and as previously reported [45]. Pictures were taken with a Nikon Eclipse microscope (Nikon Instruments Inc., NY, USA) with a 1394 color digital camera (Scion corp. Model 1310, NY, USA), and Image J software (Version 1.34s Wayne Rasband, Java 1.5.0_19) was used to analyze the pictures.

### Biomechanical testing

Biomechanical testing was performed using methods previously described [43]. At the time of testing, female reproductive tissues were carefully harvested, removing all musculature and surrounding soft tissue, and hydrated in phosphate buffered saline (PBS) (N = 12 animals of each experimental group). Orientation of the cervix was noted to ensure consistency throughout the biomechanical experiments. The cervix was dissected free of any extra soft tissue and the uterus and vagina were carefully removed. The cervix was laid flat to expose the lumen. The ends were affixed between two pieces of sandpaper for gripping, such that a uniaxial tensile load on the grips would simulate dilation of the cervical canal (loading occurred perpendicular to the proximal-distal direction). The prepared sample was continually immersed in PBS until the start of mechanical testing. A custom laser device was used to measure the cross sectional area at a minimum of two locations, which took less than 60 seconds [46]. The cervix was then placed in custom fixtures to grip it at both ends. The cervix was then tested under uniaxial tension using an Instron 5848 testing system (Instron Corp., Norwood, MA). The testing protocol consisted of a preload of 0.005N followed by a hold of 5 minutes and then a ramp to failure at a rate of 1mm/minute. The entire test was performed in a saline bath at room temperature. The location of failure was recorded for each sample. Samples were excluded from further analysis if failure did not occur within the mid-substance of the tissue.

### Statistical analysis

Statistical analyses were performed for all experiments with the GraphPad Prism Software (Version 4.0, La Jolla, CA, USA). For data that were normally distributed, an unpaired t-test was used. If data were not normally distributed, then the unpaired t-test with Welch’s correction was used. For biomechanical testing, t-test analyses were used to compare between groups for mechanical parameters. One-way ANOVA with Bonferroni-corrected post hoc tests were used to evaluate differences in fiber re-alignment. P<0.05 was considered to be statistically significant. P<0.1 was considered to be a trend.

## Results

### *G*. *vaginalis* colonization of the CV space of timed-pregnant CD-1 mice

**Fig 1:**
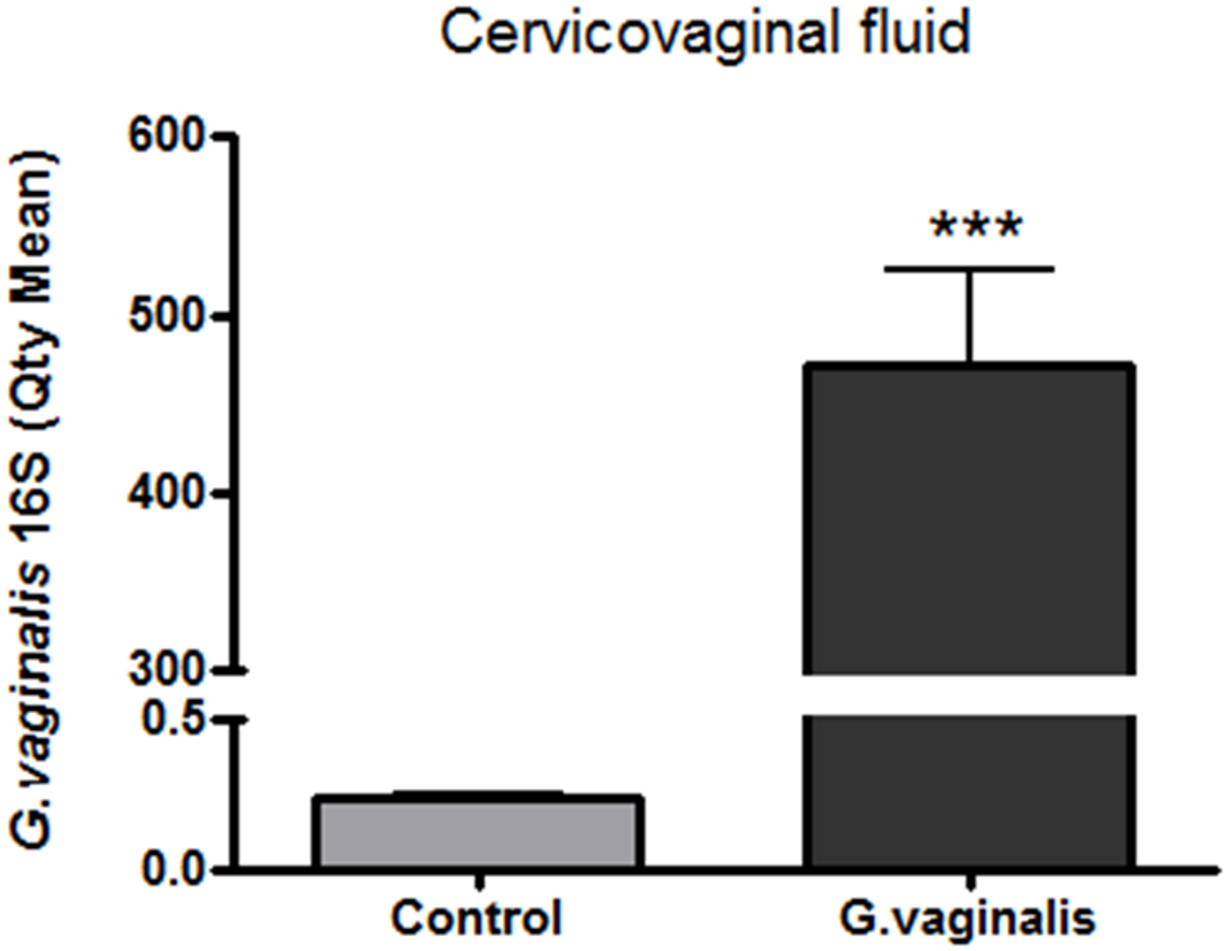
*G*. *vaginalis* colonization of the CV space of timed-pregnant CD-1 mice. Quantification of the 16S gene of *G*. *vaginalis* in the CVF of animals inoculated with 5X10^8^ CFU/mL, was performed via qPCR using a specific *G*. *vaginalis* 16S probe. Graphs shows the average quantity mean detected by qPCR of N = 8 Control and N = 12 *G*. *vaginalis* group. T-test analyses with Welch’s correction between these groups was performed (***p<0.0001). Values are mean ± SD.

*G*. *vaginalis* successfully colonized the CV space (Fig 1, p<0.0001) and was not detected in the uterus or placentas (S1 Methods; S1–S3 Figs). The most effective colonization was achieved using a single dose of 5x10^8^ CFU/mL of bacteria inoculated into the CV space for two consecutive days. In this study, we confirmed that live *G*. *vaginalis* was present in the CV space 48 hours after first inoculation (S4 Fig). Importantly, *G*. *vaginalis* colonization did not affect the litter size or the pup weight (S5 Fig). Animals treated with 5x10^8^ CFU/mL *G*. *vaginalis* had a PTB rate that ranged from 0 to 20 percent in three independent experiments (3 out of 12, 1 out of 10 and 0 out of 12 animals) delivered before E18,which is our metric standard to define PTB (data not shown). The CV space of the animals treated with 5x10^10^ CFU/mL *G*. *vaginalis* were adequately colonized (S6 Fig) and had a PTB rate of 0 percent (0 out of 10) (data not shown).

### Elevated inflammation in the cervix of *G*. *vaginalis* colonized animals

**Fig 2:**
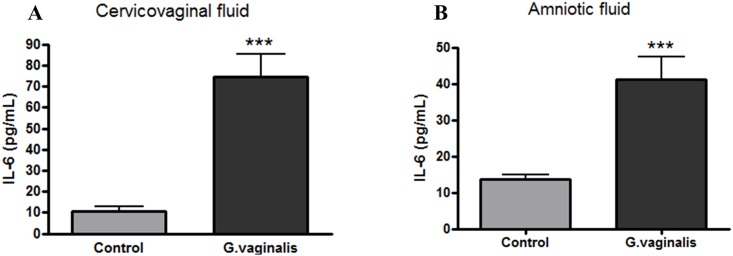
*G*. *vaginalis* increases levels of IL-6 in the cervicovaginal space and amniotic fluid. Levels of IL-6 in the CVF (**A)** and in the AF **(B)** were measured via ELISA. T-test analysis with Welch’s correction was performed to determine statistical significance between two groups (***p = 0.0007 in the CVF and ***p = 0.0008 in the AF analysis) (N = 8 Control and N = 12 *G*. *vaginalis* group). Values are mean ± SD.

**Fig 3:**
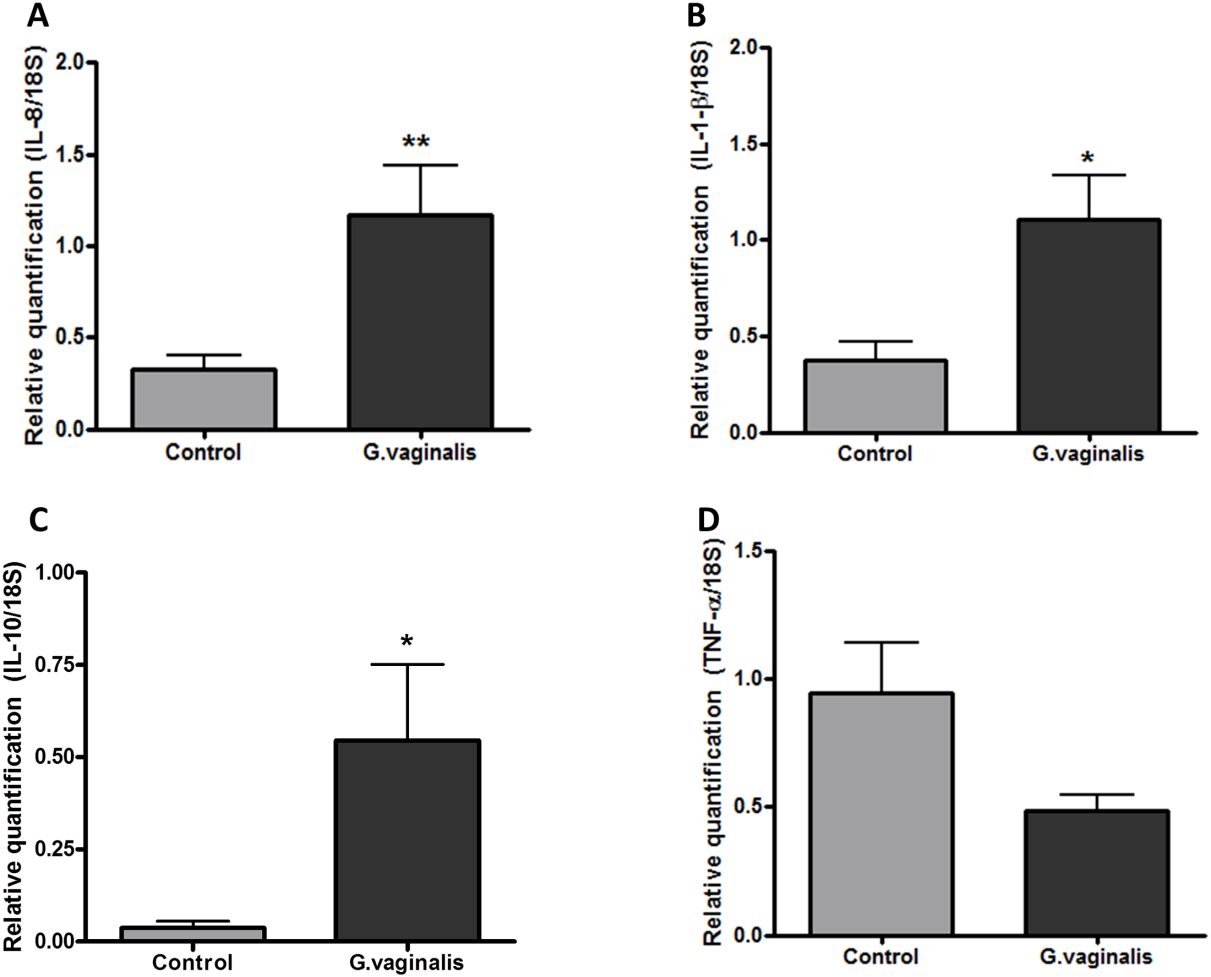
Increased gene expression of IL-1β, IL-8 and IL-10 in the cervix of *G*. *vaginalis* colonized animals. Gene expression levels of IL-8 (A), IL-1-β (B), IL-10 (C), and TNF-α (D) were measured by QPCR. T-test with Welch’s correction was performed in each group (N = 8 Control and N = 11 *G*. *vaginalis* group) (**IL-8 (p = 0.0055),*IL-1-β (p = 0.0120) and *IL-10 (p = 0.0140)). Values are mean ± SD.

In order to determine if *G*. *vaginalis* colonization of the CV space results in a local inflammatory response, we assessed IL-6 protein levels as a marker of generalized inflammation. IL-6 was significantly increased in the CVF of animals inoculated with *G*. *vaginalis* in comparison to the samples in the control group (Fig 2A, p = 0.0007). We also observed a significant increase in IL-6 in the amniotic fluid (Fig 2B, p = 0.0008) of animals colonized by *G*. *vaginalis*, despite the absence of ascending bacteria into the fetal membranes, placenta or uterus. Additionally, we quantified the gene expression of other known pro-inflammatory cytokines and chemokines in the cervix including TNF-α, IL-10, IL1β and IL-8. Cervical gene expression of IL-8 (p = 0.0055), IL-1β (p = 0.0120) and IL-10 (p = 0.0140) were significantly enhanced (Fig 3A, 3B and 3C respectively). TNF-α was not significantly altered (Fig 3D, p = 0.0842).

### Colonization of the CV space with *G*. *vaginalis* induces cervical remodeling

**Fig 4:**
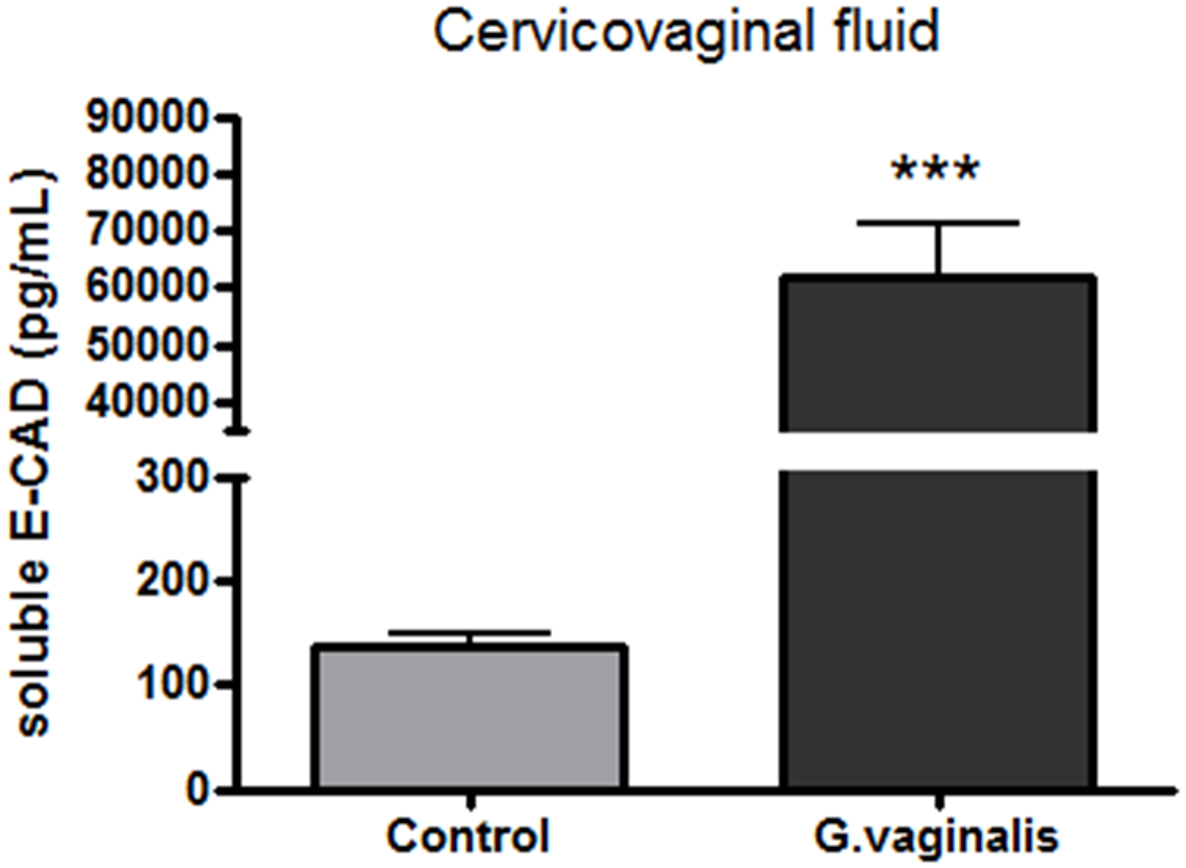
*G*. *vaginalis* increases soluble E-cadherin in the CV space. Levels of soluble E-cadherin were measured by ELISA. T-test analysis with Welch’s correction was calculated for significance of protein expression between the groups (***p<0.0001). Values are mean ± SD.

As we have shown previously that increased seCAD is a molecular marker of cervical epithelial barrier disruption, [47] we assessed if seCAD was altered in this model. Levels of seCAD were significantly increased in dams colonized with *G*. *vaginalis* compared to controls (Fig 4, p<0.0001). In addition to seCAD, we measured the gene expression of LOX, HAS-2, Tff-1 and SPINK-5 which have all been previously reported to be involved in cervical remodeling [48]. We observed increased expression of Tff-1 (Table 1, p = 0.026), whereas the gene expression levels of SPINK-5 (Table 1, p = 0.080), HAS-2 (Table 1, p = 0.076) and LOX (Table 1, p = 0.3625) were not significantly different.

**Table 1:** Increased expression of Tff-1 in the cervix of *G*. *vaginalis* colonized animals.

Protein	Fold-change	p-values
**Tff-1**	3*	**p = 0.03**
**SPINK-5**	**1**	**p = 0.08**
**HAS-2**	**1**	**p = 0.08**
**LOX**	**-1**	**p = 0.36**

The expression levels of proteins associated with cervical remodeling Tff-1, SPINK-5, HAS-2, and LOX were measured by qPCR. T-test with Welch’s correction test was performed between each group (N = 8 Control and N = 11 *G*. *vaginalis* group). Asterisk indicates statistical significance with a p-value <0.05.

**Fig 5:**
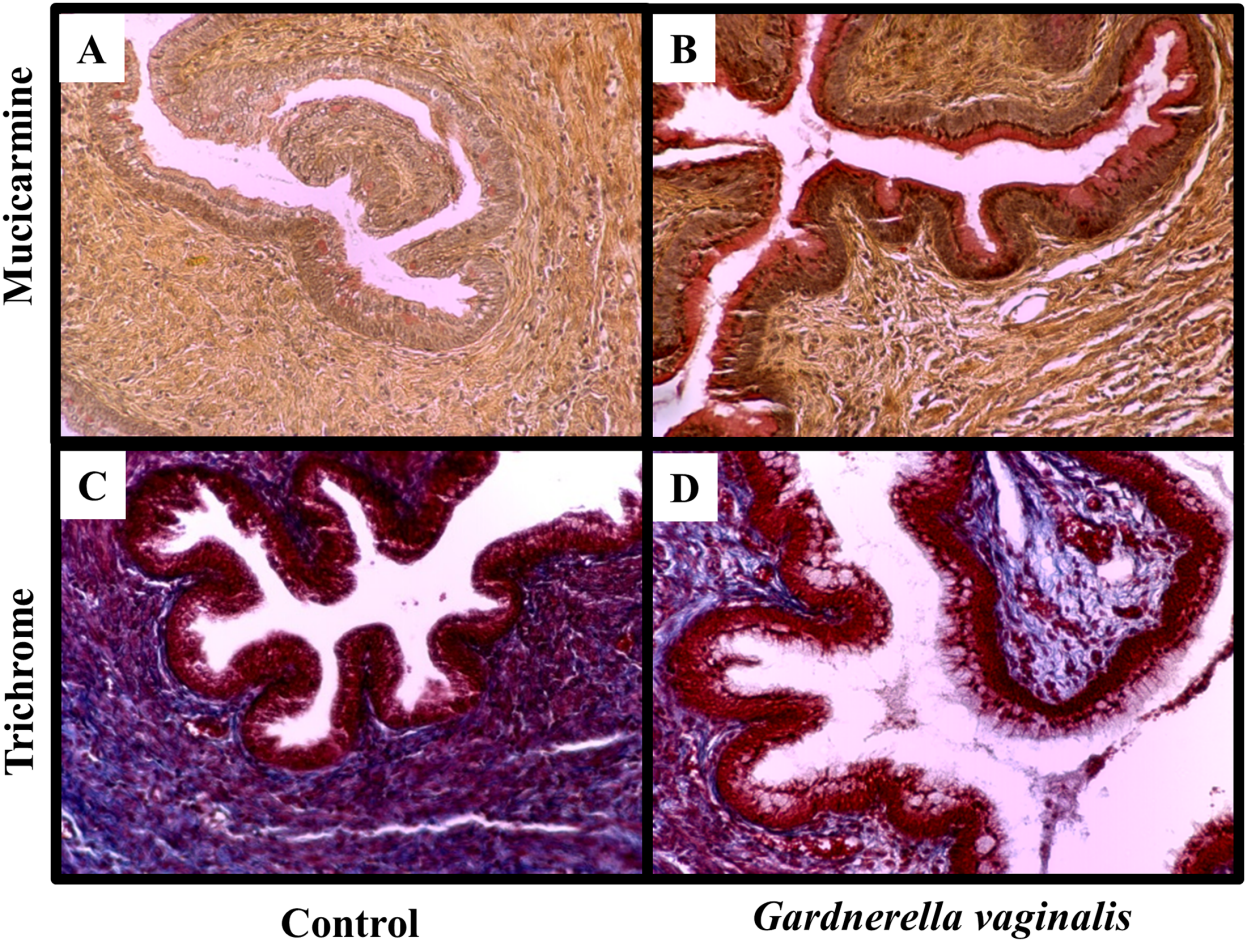
*G*. *vaginalis* colonization increased expression of mucin and decreased collagen dispersion within cervical tissues. Representative cervical sections from Control (A, C) or *G*. *vaginalis* (B, D) treated animlas (N = 4 in each group). Cervices were stained with mucicarmine (A, B) for analysis of mucin production while trichrome stain (C, D) shows collagen dispersion. Pictures were taken at a 10X magnification. Trichrome stains collagen blue, muscle fibers red and nuclei black-blue. Mucicarmine stains mucin pink/red, the nuclei blue and any other tissue component yellow.

Prior work has demonstrated that histological changes within the cervix are consistent with cervical remodeling [49, 50]. Mucicarmine and trichrome stainings were performed to assess for the presence of mucin and collagen in cervices of *G*. *vaginalis* colonized compared to control animals (Fig 5). We observed increased expression of mucin in the cervices from dams colonized with *G*. *vaginalis* compared to controls, consistent with cervical ripening (Fig 5A and 5B). Additionally, we observed a dispersion of collagen fibers in animals colonized with *G*. *vaginalis* in comparison to animals treated with sugar water (Fig 5C and 5D).

### *G*. *vaginalis* colonization alters the cervical biomechanics

**Fig 6:**
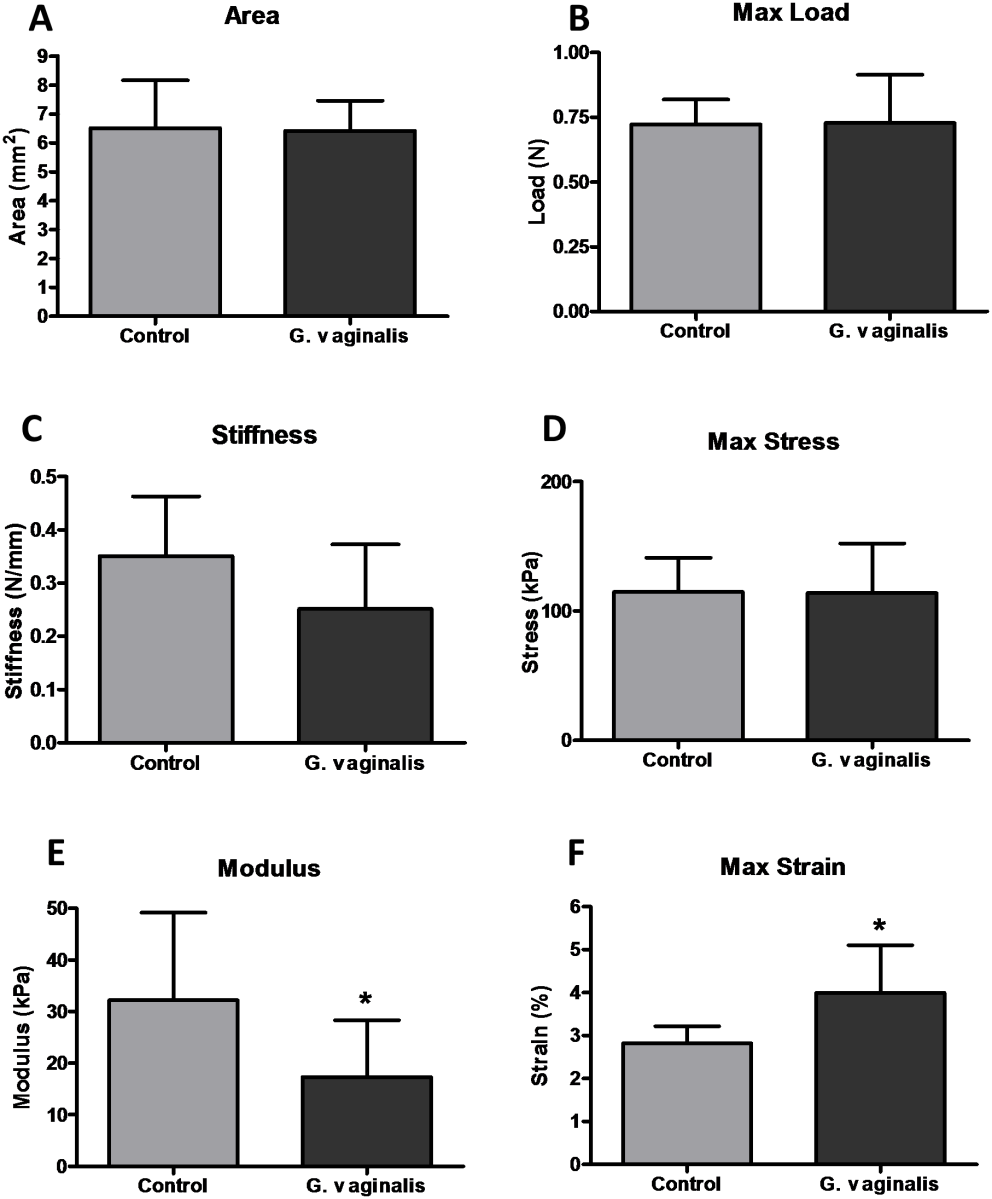
Mechanical properties of *G*. *vaginalis* colonized cervices. Area (A), Max Load (B), Stiffness (C), Max Stress (D), Modulus (E), and Max Strain (F) are shown for both control and *G*. *vaginalis* colonized cervices. All data is presented as means with standard deviations and significance noted at p< 0.05, (n = 10–11 in each group).

Using previously described [43] techniques and metrics, we found that colonization with *G*. *vaginalis* demonstrated a decrease in modulus (Fig 6E, p< 0.05) as well as an increase in maximum strain (Fig 6F, p< 0.05) but no change in tissue cross-sectional area (Fig 6A), maximum load (Fig 6B), stiffness (Fig 6C, p<0.1) or maximum stress (Fig 6D). No difference in collagen fiber re-alignment was observed during cervical mechanical testing between groups (S1 Methods; S7 Fig).

## Discussion

As bacterial infection is thought to be one of the predominate factors associated with spontaneous PTB [1, 2] this study has provided novel insight into the role of cervicovaginal (CV) bacteria in the CV space and, specifically, on cervical function. The results from this study suggest that *G*. *vaginalis*, the most common bacteria associated with BV [4, 5], has the ability to initiate cervical remodeling through multiple biological, molecular and biomechanical mechanisms. In this study, we successfully generated a novel pregnant mouse model with a CV space colonized with *G*. *vaginalis*. This new model allowed us to study the effects of this bacterium within the CV space and its association with premature cervical remodeling. This study provides evidence that *G*. *vaginalis* colonization, localized to the CV space, was able to alter cervical function through multiple biological mechanisms including activating a cervical immune response, initiating cervical remodeling and modifying the material biomechanical properties of the cervix. Therefore, these results suggest that the presence of *G*. *vaginalis* within the CV space during pregnancy has the ability to directly alter many of the biological mechanisms regulating cervical remodeling and, hence, could contribute to the pathogenesis of PTB.

A previous study investigating the effects of *G*. *vaginalis* in the CV space of a non-pregnant mouse model showed that *G*. *vaginalis* was able to replicate within the CV space and ascend into the uterine horns [44]. Additionally, this same study showed that CV colonization of *G*. *vaginalis* resulted in many of the hallmark symptoms typically associated with clinical BV such as increased sialidase activity and vaginal epithelial exfoliation similar to phenotypes observed in clue cells in BV cases [44]. Similarly, in our *G*. *vaginalis* colonized pregnant mouse model, we observed high levels of *G*. *vaginalis* 16S in the CV space 48 hours post inoculation. It is important to point out that *G*. *vaginalis* inoculated into the CV space remained alive 48 hours post inoculation as evidenced by the growth of *G*. *vaginalis* colonies from CVF lavages (S6 Fig). *G*. *vaginalis* 16S was not detected within the uterus, placenta or fetal membranes suggesting that, in our model, *G*. *vaginalis* primarily colonizes the CV space with no ascension into the uterus. In contrast, it has been shown that *G*. *vaginalis* is capable of ascending into the uterus of a non-pregnant mouse model [44]. The ability of *G*. *vaginalis* to ascend into the uterus in the non-pregnant model but not in our pregnant model is biologically important. Due to the presence of increased cervicovaginal mucus known to be associated with pregnancy, it is biologically plausible to suggest that the presence of increased cervical mucus (and its associated mucosal immunity) may play a significant role in preventing the ascension of *G*. *vaginalis* into the uterus. Indeed, it has been observed that non pregnant mice have ascending bacteria, but this does not occur in pregnant animals as we have demonstrated. The inability of bacteria to ascend in the pregnant state may be due to expression changes of toll-like receptors and anti-microbial peptides (eg mucin) on the cervix during pregnancy [51]. The absence of detectable bacteria in the uterus correlates with the fact that *G*. *vaginalis* colonization did not affect litter size or pup weight (S4 Fig). To our knowledge this study is the first to show *G*. *vaginalis* colonization in pregnant mice.

The varied PTB rate animals treated with *G*. *vaginalis* may be attributed to the out-bred genetic background of our mouse model which inherently adds to a variable outcome and response to the colonization of *G*. *vaginalis*. Studies showing varying associations between BV treatment and PTB rates [4] may suggest that, in human pregnancy, *G*. *vaginalis* alone may not be sufficient to cause sPTB. Instead, *G*. *vaginalis* in combination with other BV-associated (or other non-BV) bacteria may be needed in order to initiate a more consistent preterm birth phenotype. Additionally, it is possible that the exposure time of *G*. *vaginalis* in the CV space could have significant effects on the cervix. In our model the animals were only exposed to *G*. *vaginalis* beginning on embryonic day 12, for a maximum time ranging from 48 hours to 5 days. Therefore, we cannot rule out the duration of exposure to *G*. *vaginalis* as being a contributing factor to the pathogenesis of preterm birth. Finally, it is important to note that the mouse epithelium is keratinized in contrast to the human, therefore *G*. *vaginalis* colonization and adherence might be different in our mouse model as compared to the human population.

The increased expression of IL-6 in the CVF of animals colonized with *G*. *vaginalis* indicated that the presence of this bacterium was able to initiate a localized immune response within the CV space. Interestingly, even in the absence of ascending bacteria, elevated IL-6 in the AF of animals inoculated with *G*. *vaginalis* suggests that increased cytokines localized to the CV space might have the ability to further activate an inflammatory response within the uterine cavity. Furthermore, when we analyzed the gene expression of these cytokines/chemokines in the cervix of *G*. *vaginalis* colonized animals, we observed an increase in PTB-associated cytokines [3, 52] such as IL-8, IL-10 and IL-1β. In a reported non-pregnant model, *G*. *vaginalis* colonization showed no histological inflammation [44], however cytokine and chemokine expression levels were not assessed after CV infection. Therefore, our results provide pertinent information about the inflammatory pathways induced by *G*. *vaginalis* colonization of the CV space during pregnancy. Our study is the first to show that *G*. *vaginalis* has the ability to induce an inflammatory response within the CV space of pregnant mice and it is biologically plausible that this inflammation may be capable of altering cervical function and integrity.

Previous work from our laboratory has demonstrated that an inflammatory insult leads to breakdown of the cervical epithelial barrier [47]. These observations are also present in other mucosal epithelial tissues such as the gut [53–59]. In the gut, inflammation leads to the activation of matrix metalloproteases (MMPs) that lead to disruption of the epithelial junctional proteins including epithelial-cadherin (e-cadherin) [53]. Specifically, MMPs and other serine proteases cleave the extracellular domain of e-cadherin resulting in the release of soluble e-cadherin (seCAD) into the extracellular spaces. Thus, in the presence of *G*. *vaginalis* colonization, increased seCAD in the CVF indicates a breakdown of the adherens junctions within the cervical epithelial barrier, as we have demonstrated in *in vitro* studies with cervical epithelial cells [47].Therefore, in our pregnant animal model, the fact that *G*. *vaginalis* colonization leads to increased levels of seCAD suggests that inflammation within the CV space has the ability to initiate the breakdown of the cervical epithelial barrier leading to cervical remodeling.

Based on these results, we aimed to further demonstrate that colonization of the CV space with *G*. *vaginalis* results in cervical remodeling. Previous work has demonstrated numerous molecular markers associated with cervical ripening and remodeling in different embryonic stages of mouse pregnancy [32]. To confirm cervical remodeling, we assessed LOX, Tff-1 and SPINK-5 gene expression as they have been reported to be associated with cervical ripening and dilation [32, 35]. Interestingly, increases in Tff-1 have been previously associated with increased internalization of e-cadherin, a process that occurs upon cleavage of the extracellular domain of e-cadherin resulting in an elevation of seCAD, as was observed in this study [60]. Thus, increased Tff-1 gene expression levels in the cervix along with the significantly elevated levels of seCAD in the CVF, provide evidence that *G*. *vaginalis* has the ability to alter the process of cervical remodeling. There are characteristic histological changes of the mouse cervix indicative of cervical remodeling; especially as parturition approaches there is evidence of collagen rearrangement and a decrease in cervical stiffness [32]. In addition to the observed changes in gene expression, mucicarmine and trichrome staining showed an increase in mucin expression as well as a dispersion of collagen fibers in the cervix of animals colonized with *G*. *vaginalis* providing additional evidence of cervical remodeling. While it is unknown if the alterations in histological cervical remodeling or an activated immune response occurs first, it is interesting to note, that increased mucin production has been linked to activation of the innate immune system as a response to bacterial infection [61]. Therefore, it is plausible to hypothesize that mucin is increased, in part, through the host’s biological mechanisms to protect the cervical epithelial cells from *G*. *vaginalis* infection. These results agree with previous studies showing an activated inflammatory response causes an increase in histological mucin expression and collagen dispersion [45, 50, 62]. While the exact pathological mechanisms leading to histological cervical remodeling remain unclear, the results from this study provide evidence that *G*. *vaginalis* has the ability to increase cervical remodeling.

Since both molecular and histological differences were observed in the cervix due to colonization of the CV space with *G*. *vaginalis*, cervical biomechanical parameters were also assessed. There was a significant decrease in tissue modulus of cervices colonized with *G*. *vaginalis*, when compared with the controls, indicating a change in its inherent material response. Concomitantly, a trend towards decreased stiffness of the cervical tissue points towards a structural (size) increase. This indicates a clear differential mechanical response of the murine cervix to colonization with *G*. *vaginalis*. Further, we have previously observed similar material and structural changes in the normal pregnant cervix immediately prior to parturition (E18.5). Interestingly, although the modulus values fall to similar levels as those observed in the normal E18.5 cervices, stiffness of the *G*. *vaginalis* samples remains appreciably higher. This finding suggests that some of the mechanical mechanisms contributing to cervical remodeling are different in animals colonized with *G*. *vaginalis* opposed to term parturition [36]. It is important to note that some of the observed mechanical changes such as modulus and stiffness in the *G*. *vaginalis* cervices could indicate more rapid cervical remodeling. In our model, colonization of the CV space with *G*. *vaginalis* provides evidence that cervical softening is occurring faster/earlier in comparison to our control group, as well as, in normal gestation [36, 40].

One limitation of this animal model is that, unlike the human, mice are quadrupedal not bipedal. The load of pregnancy would be divergently distributed in the mouse compared to the human and could affect cervical biomechanics. Despite this limitation, in the future it is imperative to continue to define how both structural and material mechanical properties of the cervix work in tandem with other biological and mechanical factors to regulate both term and preterm birth.

We did not observe drastic differences in collagen fiber re-alignment between cervices colonized with *G*. *vaginalis* versus control (S5 Fig.) indicating that the decrease in stiffness and modulus of cervical tissue in the *G*. *vaginalis* samples was not due to any reorganization of the load-bearing response of cervical collagen fibers. However, other factors such as collagen cross-linking, density of collagen fibers, or change in collagen fiber diameter could also explain the observed decrease in tissue mechanical properties [36, 63]. Both the dispersed collagen observed histologically combined with the increase of mucin expression could indicate other mechanisms of cervical remodeling through activation of immunological pathways.

Collectively, the results of this study show that colonization of *G*. *vaginalis* in the cervicovaginal space of pregnant mice has the ability to significantly alter cervical function. By evaluating multiple biological mechanisms known to be associated with the cervical remodeling process, we demonstrated that colonization of *G*. *vaginalis* in the cervicovaginal space can induce local inflammation, damage the cervical epithelial barrier, initiate cervical remodeling and alter the biomechanical characteristics of the cervix. The ability of *G*. *vaginalis* to induce these molecular, immune and cellular changes suggests that this bacterium could play a mechanistic role in sPTB in which cervical remodeling is the initiating event. Additionally, this study demonstrates the feasibility of mimicking the human CV microbiota in a pregnant mouse model. As shown in a recent study, *G*. *vaginalis* colonization has implications for other lower genitourinary tract conditions, such as the case of recurrent *E*.*coli* infections in the bladder [64]. By elucidating the mechanisms by which *G*. *vaginalis* alters the epithelium, underlying tissue and immune responses in the CV space, we might provide increased understanding of conditions associated with *G*. *vaginalis* such as HIV [65–70], UTI [64], recurrent pregnancy loss and preterm birth [71]. These findings have broader implications. An increased understanding of the role of the cervicovaginal microbiome and how they might mitigate or modify molecular, biomechanical and immune function in the CV space will be essential to developing future therapeutic options for preventing sPTB.

## Supporting information

S1 Fig Presence of *G*. *vaginalis* in the CVF. gDNA from the CVF was used with a *G*. *vaginalis* specific primer set to amplify *G*. *vaginalis* via PCR. PCR reactions were run on a 1% agarose gel with ethidium bromide and exposed to UV light to capture DNA bands. *G*. *vaginalis* positive bands were expected at an amplicon of 206 bp. As a positive control we included a PCR sample of gDNA isolated directly from *G*. *vaginalis* cultures. To determine the PCR product sizes we included wells with 1Kb and 100 bp ladders on each side of the gel.
(TIF)

S2 Fig Presence of *G*. *vaginalis* in the uterus. gDNA from the uterus was used with a *G*. *vaginalis* specific primer set to amplify *G*. *vaginalis* via PCR. PCR reactions were run on a 1% agarose gel with ethidium bromide and exposed to UV light to capture DNA bands. *G*. *vaginalis* positive bands were expected at an amplicon of 206 bp. As a positive control we included a PCR sample of gDNA isolated directly from *G*. *vaginalis* cultures. To determine the PCR product sizes we included wells with 1Kb and 100 bp ladders on each side of the gel.
(TIF)

S3 Fig Presence of *G*. *vaginalis* in placenta. gDNA from the placenta was used with a *G*. *vaginalis* specific primer set to amplify *G*. *vaginalis* via PCR. PCR reactions were run on a 1% agarose gel with ethidium bromide and exposed to UV light to capture DNA bands. *G*. *vaginalis* positive bands were expected at an amplicon of 206 bp. As a positive control we included a PCR sample with gDNA isolated directly from *G*. *vaginalis* cultures. To determine the PCR product sizes we included wells with 1Kb and 100 bp ladders on each side of the gel.
(TIF)

S4 Fig *G*. *vaginalis* live bacteria in the CVF 48 hours post inoculation. Tryptic Soy Agar plates supplemented with 5% defibrillated rabbit blood were inoculated with 50μL of CVF collected from mice 48 hours post-inoculation and incubated for 72 hours in an anaerobic jar at 37°C and 5% CO2. After incubation, the numbers of colonies were counted on each plate.
(TIF)

S5 Fig Treatment with *G*. *vaginalis* does not affect pup weight or litter size. Dams treated with sugar water or 5X10^8^ CFU/mL of *G*. *vaginalis* were allowed to deliver (N = 8 in each group). The individual pup weights (A) and the number of pups per litter (B) were recorded. T-test with Mann-Whitney nonparametric correction analysis was performed to determine statistical significance between the two groups (pup weight: p = 0.8785 and Litter size: p = 0.6454). Values are mean ± SD.
(TIF)

S6 Fig *G*. *vaginalis* colonization of the CV space of timed-pregnant CD-1 mice using a higher bacterial dose. Quantification of the 16S gene of *G*. *vaginalis* in the CVF of animals inoculated with 5X10^10^ CFU/mL was performed via qPCR using a specific *G*. *vaginalis* 16S probe. Graphs shows the average quantity mean detected by qPCR of N = 10 Control and N = 10 *G*. *vaginalis* group. T-test analyses with Welch’s correction between these groups was performed (p = 0.0002). Values are mean ± SD.
(TIF)

S7 Fig Collagen fiber re-alignment measured by polarized light analysis during cervical mechanical testing. Representative plots of polarized light analysis at toe, end of toe, 45% of maximum load, and 90% of maximum load. The control group is shown on the left (A) and *G*. *vaginalis* colonized cervices are shown on the right (B). Lines represent significance of p< 0.05 (n = 10–11).
(TIF)

S1 Methods *G*. *vaginalis* PCR and cervix collagen fiber alignment methodology. (PDF)
