# Supplementary figures and images for: Colonization of the cervicovaginal space with Gardnerella vaginalis leads to local inflammation and cervical remodeling in pregnant mice
Source: PLoS One. 2018 Jan 18;13(1):e0191524. doi: 10.1371/journal.pone.0191524 (PMC5773211; doi:10.1371/journal.pone.0191524)

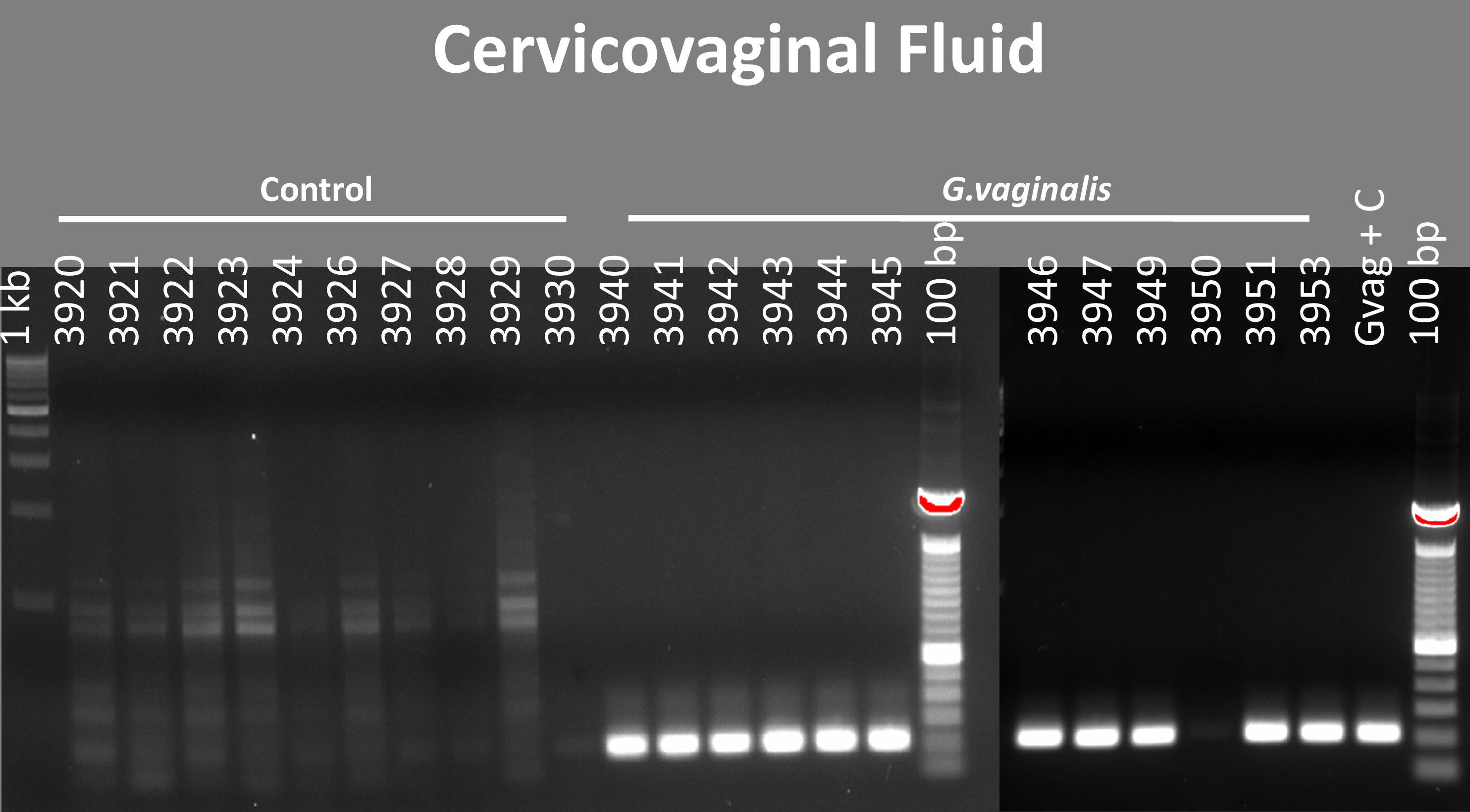

Supplement: S1 Fig — gDNA from the CVF was used with a G. vaginalis specific primer set to amplify G. vaginalis via PCR. PCR reactions were run on a 1% agarose gel with ethidium bromide and exposed to UV light to capture DNA bands. G. vaginalis positive bands were expected at an amplicon of 206 bp. As a positive control we included a PCR sample of gDNA isolated directly from G. vaginalis cultures. To determine the PCR product sizes we included wells with 1Kb and 100 bp ladders on each side of the gel. (TIF) [file pone.0191524.s001.tif]

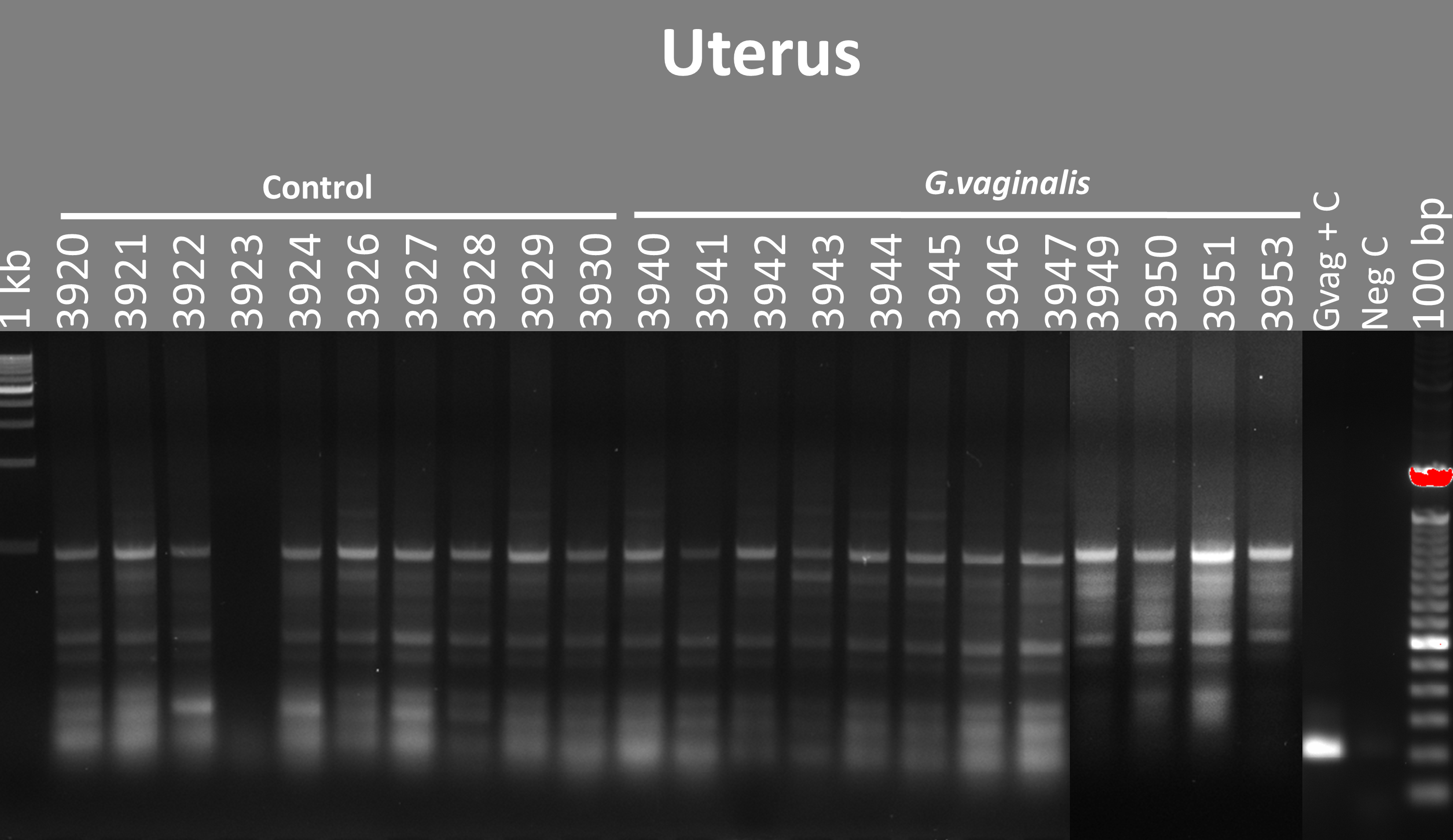

Supplement: S2 Fig — gDNA from the uterus was used with a G. vaginalis specific primer set to amplify G. vaginalis via PCR. PCR reactions were run on a 1% agarose gel with ethidium bromide and exposed to UV light to capture DNA bands. G. vaginalis positive bands were expected at an amplicon of 206 bp. As a positive control we included a PCR sample of gDNA isolated directly from G. vaginalis cultures. To determine the PCR product sizes we included wells with 1Kb and 100 bp ladders on each side of the gel. (TIF) [file pone.0191524.s002.tif]

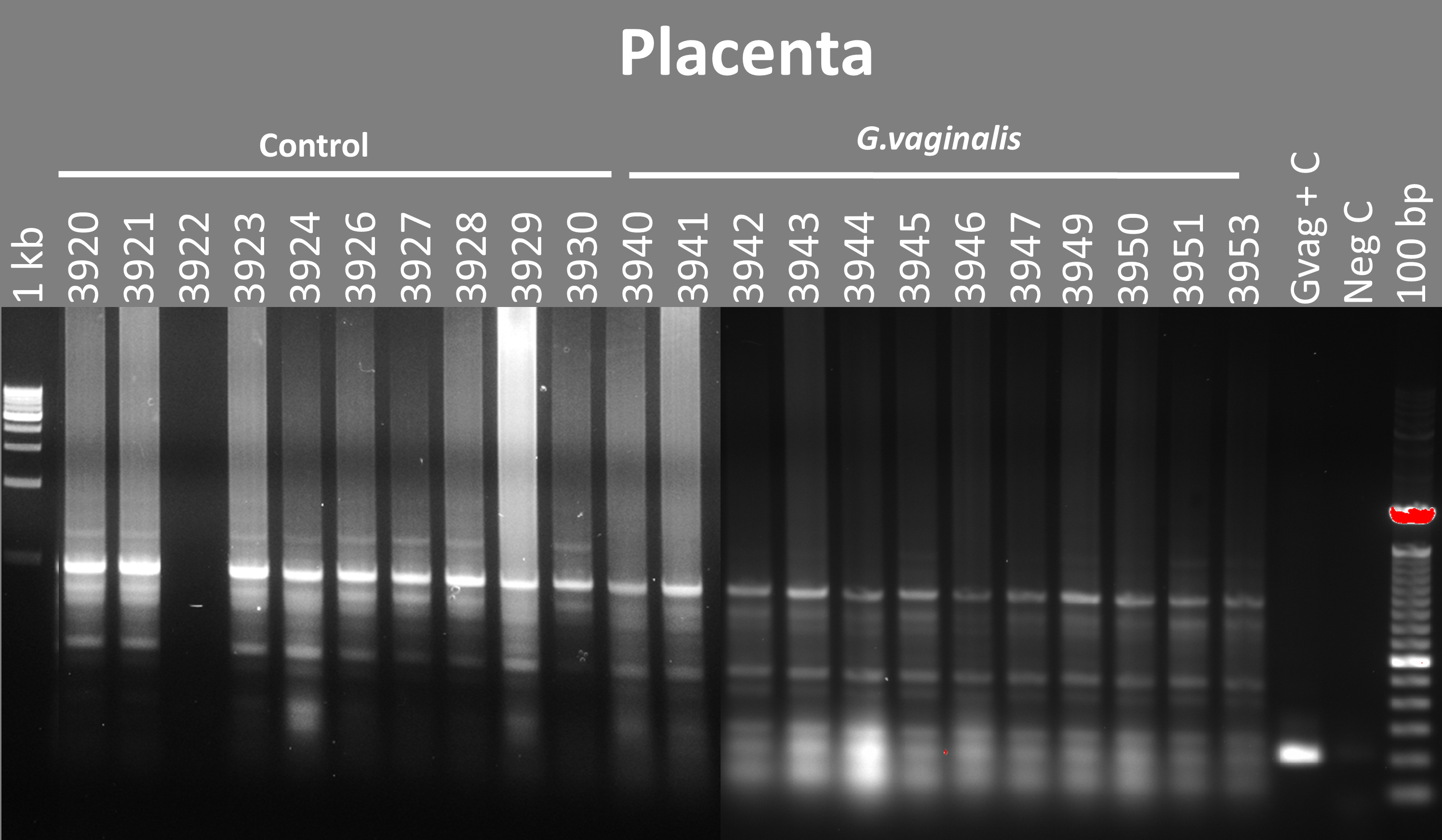

Supplement: S3 Fig — gDNA from the placenta was used with a G. vaginalis specific primer set to amplify G. vaginalis via PCR. PCR reactions were run on a 1% agarose gel with ethidium bromide and exposed to UV light to capture DNA bands. G. vaginalis positive bands were expected at an amplicon of 206 bp. As a positive control we included a PCR sample with gDNA isolated directly from G. vaginalis cultures. To determine the PCR product sizes we included wells with 1Kb and 100 bp ladders on each side of the gel. (TIF) [file pone.0191524.s003.tif]

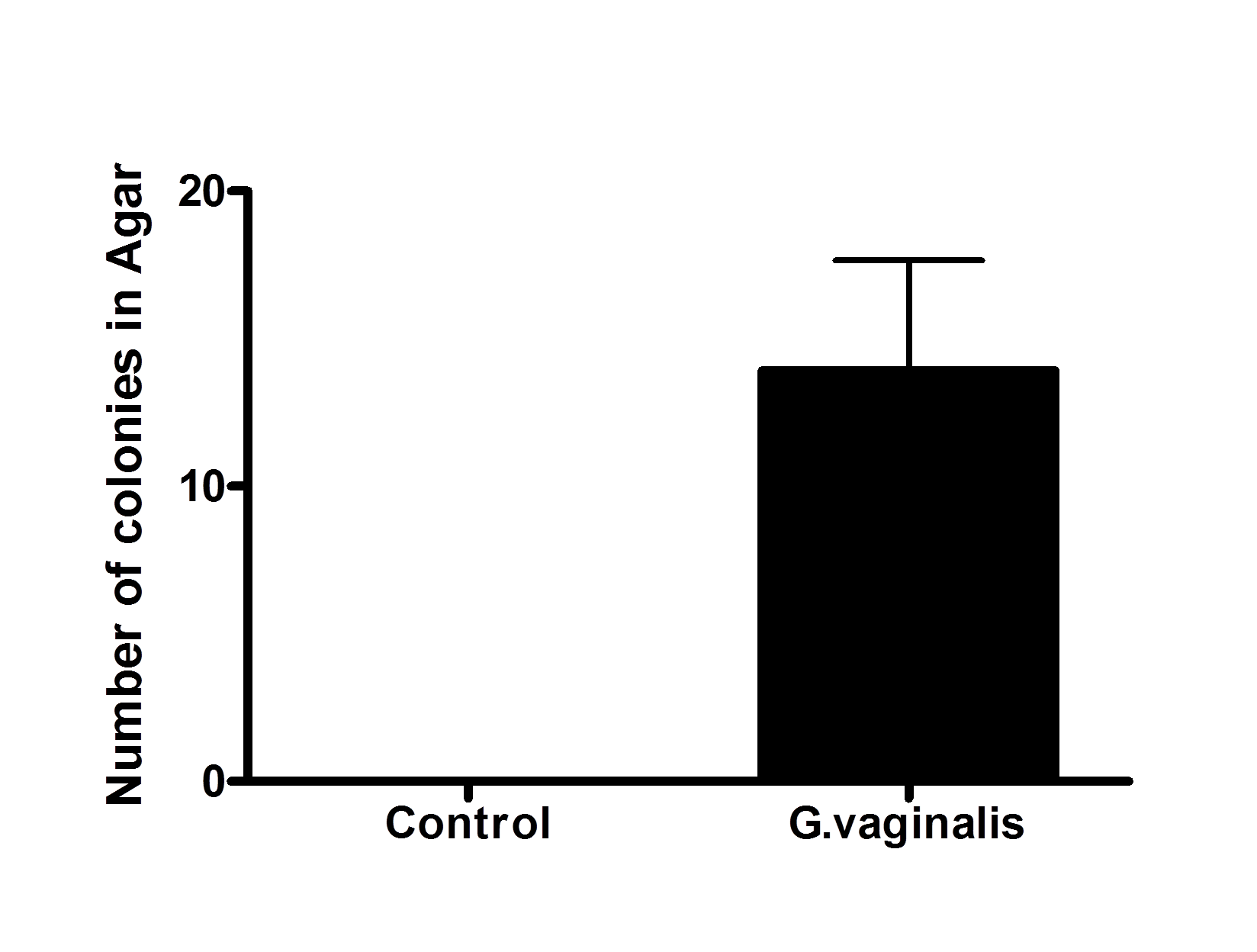

Supplement: S4 Fig — Tryptic Soy Agar plates supplemented with 5% defibrillated rabbit blood were inoculated with 50μL of CVF collected from mice 48 hours post-inoculation and incubated for 72 hours in an anaerobic jar at 37°C and 5% CO2. After incubation, the numbers of colonies were counted on each plate. (TIF) [file pone.0191524.s004.tif]

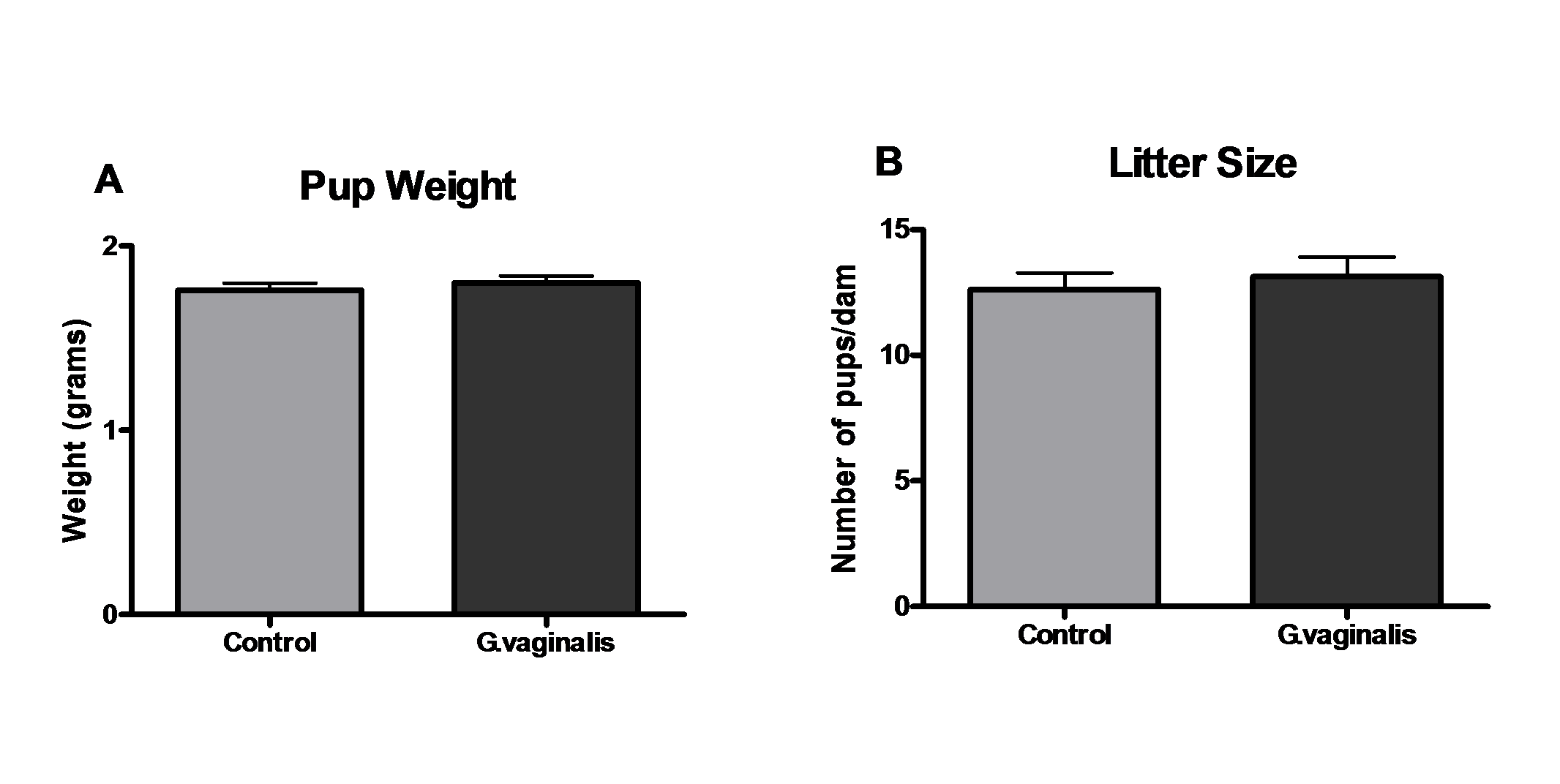

Supplement: S5 Fig — Dams treated with sugar water or 5X108 CFU/mL of G. vaginalis were allowed to deliver (N = 8 in each group). The individual pup weights (A) and the number of pups per litter (B) were recorded. T-test with Mann-Whitney nonparametric correction analysis was performed to determine statistical significance between the two groups (pup weight: p = 0.8785 and Litter size: p = 0.6454). Values are mean ± SD. (TIF) [file pone.0191524.s005.tif]

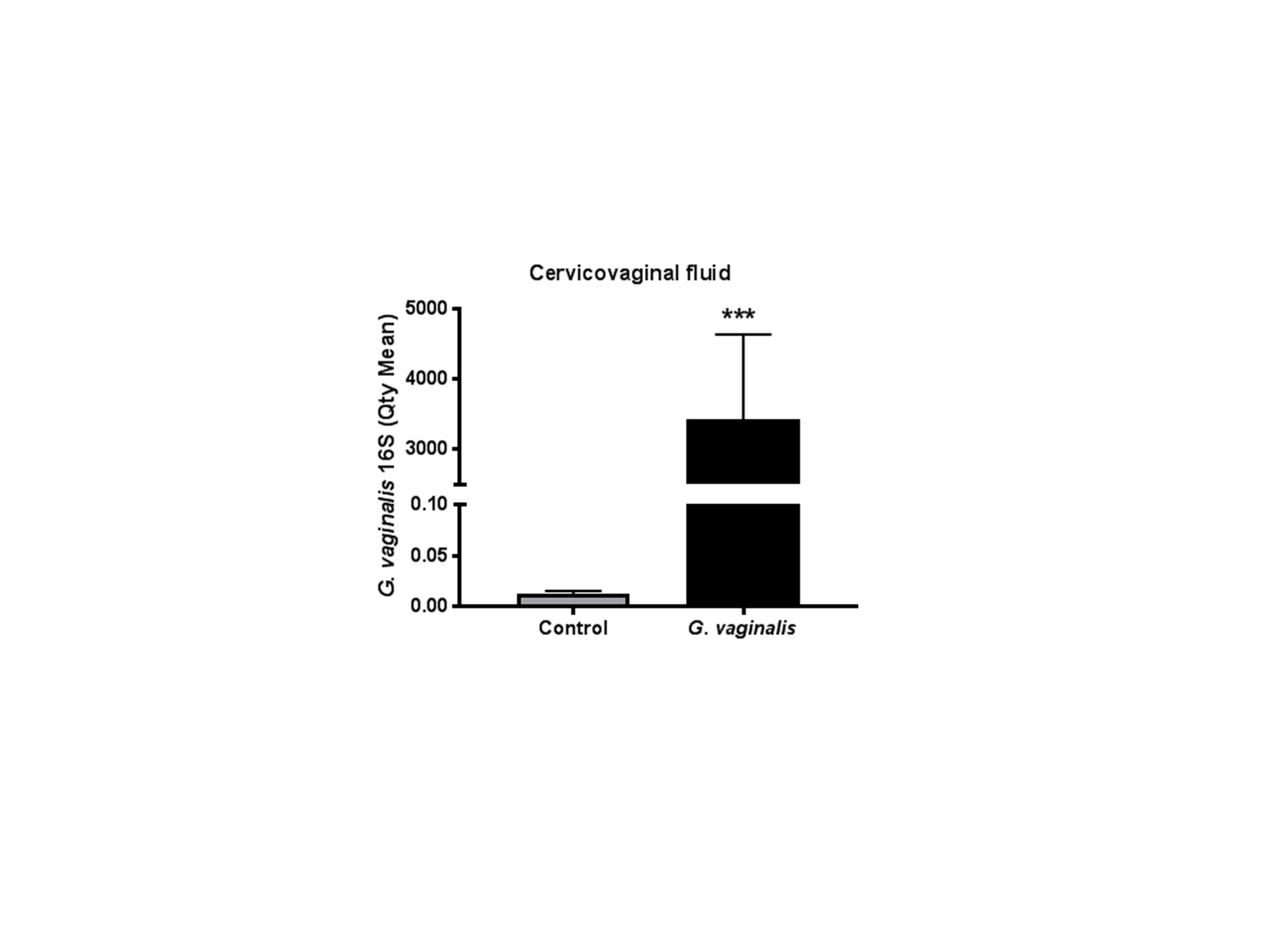

Supplement: S6 Fig — Quantification of the 16S gene of G. vaginalis in the CVF of animals inoculated with 5X1010 CFU/mL was performed via qPCR using a specific G. vaginalis 16S probe. Graphs shows the average quantity mean detected by qPCR of N = 10 Control and N = 10 G. vaginalis group. T-test analyses with Welch’s correction between these groups was performed (p = 0.0002). Values are mean ± SD. (TIF) [file pone.0191524.s006.tif]

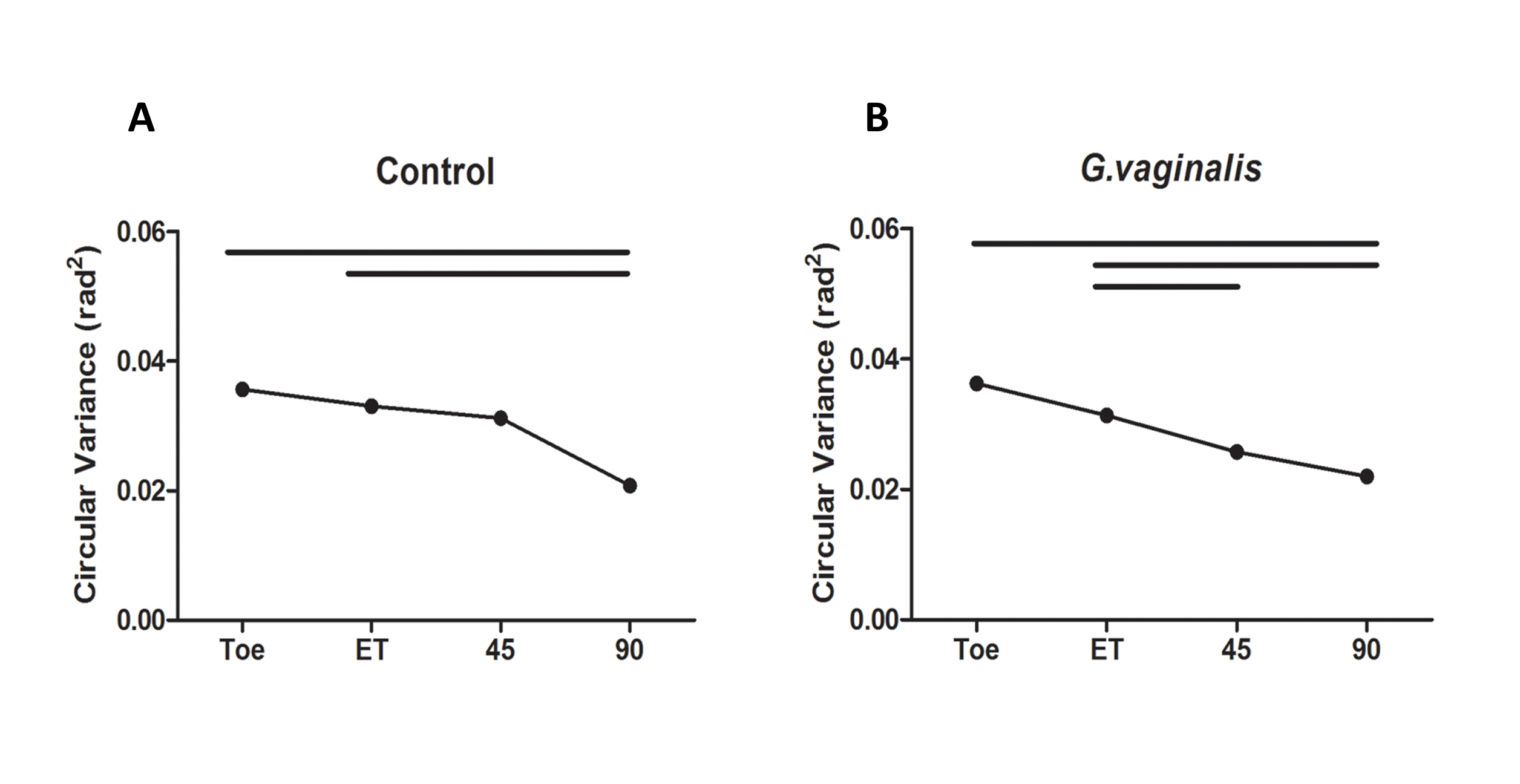

Supplement: S7 Fig — Representative plots of polarized light analysis at toe, end of toe, 45% of maximum load, and 90% of maximum load. The control group is shown on the left (A) and G. vaginalis colonized cervices are shown on the right (B). Lines represent significance of p< 0.05 (n = 10–11). (TIF) [file pone.0191524.s007.tif]
