# Supplementary material for: Colonization of the cervicovaginal space with Gardnerella vaginalis leads to local inflammation and cervical remodeling in pregnant mice
Source: PLoS One. 2018 Jan 18;13(1):e0191524. doi: 10.1371/journal.pone.0191524 (PMC5773211; doi:10.1371/journal.pone.0191524)
Supplement: S1 Methods — (PDF) [file pone.0191524.s008.pdf]

Supplementary Methods

G. vaginalis PCR

We used 0.250µg of purified Genomic DNA (gDNA) extracted from the CVF using the ZR fecal MiniPrep DNA extraction kit (Zymo Research, Irvine, CA, USA) or from placenta, uterus and fetal membranes using the DNeasy Blood and Tissue mini column DNA extraction kit (Qiagen, Germantown, MD, USA) following the manufacture's protocol. The gDNA was used to amplify a short section of *G. vaginalis* 16S in the V1 region using a custom designed primer set (primer sequence listed below) (Invitrogen). The expected amplicon product was 206 bp. The gDNA was mixed with 1X Buffer, 1U of enzyme (Denville Inc.) and 100µM of dNTPs (Thermo Fisher Scientific, Inc) following the manufacturer's instructions. *G. vaginalis* was amplified using 1µg of primers (Forward GGGCGGGCTAGAGTGCA, Reverse GAACCCGTGGAATGGGC). These primers have a melting temperature of 58°C and GC content of 65%. The PCR amplification was run with the following thermocycle conditions: 95°C for 5 min, 35 cycles at 94°C for 30 sec, 58°C for 30 sec and 72°C for 1 min, a final extension of 72°C for 5 min. The PCR reactions were carried out using the SimpliAmpThermal Cycler (Applied Biosystems) and the products were run on 1% agarose gels for 45 minutes at 150V. The agarose gels were imaged in the BioRad ChemiDoc MP Imaging System, and using the Image Lab Software (IL Version 4.1) (BioRad Laboratories, Life System Group, Hercules, CA, USA).

Cervix Collagen Fiber Alignment

Collagen fiber alignment maps of the cervix were collected throughout the mechanical testing protocol using our established integrated cross-polarizer system, as described in previous publications. [1-3] This custom system consists of a linear backlight (Dolan-Jenner, Boxborough, MA), rotating polarized sheets offset by 90° (Edmund Optics), and a camera. Custom software (National Instruments LabVIEW, Austin, TX) synchronized with analog output signals from the Instron triggered alignment maps to image capture at 7.5 second intervals. Collagen alignment was measured at four points during the mechanical test: [1]start of the toe region, defined by the first map after the hold protocol, [2,4,5] at the end of the toe region, determined by the last map before the change of slope to the linear region, [6] at 45% of the maximum load, and [3] at 90% of the maximum load. Re-alignment was defined as a significant change in circular variance between two time points.

References:

1. Dunkman AA, Buckley MR, Mienaltowski MJ, Adams SM, Thomas SJ, et al. (2013) Decorin expression is important for age-related changes in tendon structure and mechanical properties. Matrix Biol 32: 3-13.
2. Miller KS, Edelstein L, Connizzo BK, Soslowsky LJ (2012) Effect of preconditioning and stress relaxation on local collagen fiber re-alignment: inhomogeneous properties of rat supraspinatus tendon. J Biomech Eng 134: 031007.
3. Freedman BR, Sarver JJ, Buckley MR, Voleti PB, Soslowsky LJ (2014) Biomechanical and structural response of healing Achilles tendon to fatigue loading following acute injury. J Biomech 47: 2028-2034.
4. Miller KS, Connizzo BK, Feeney E, Tucker JJ, Soslowsky LJ (2012) Examining differences in local collagen fiber crimp frequency throughout mechanical testing in a developmental mouse supraspinatus tendon model. J Biomech Eng 134: 041004.
5. Miller KS, Connizzo BK, Soslowsky LJ (2012) Collagen fiber re-alignment in a neonatal developmental mouse supraspinatus tendon model. Ann Biomed Eng 40: 1102-1110.
6. Miller KS, Connizzo BK, Feeney E, Soslowsky LJ (2012) Characterizing local collagen fiber re-alignment and crimp behavior throughout mechanical testing in a mature mouse supraspinatus tendon model. J Biomech 45: 2061-2065.
